# Association of FcγRIIa R131H polymorphism with idiopathic pulmonary fibrosis severity and progression

**DOI:** 10.1186/1471-2466-10-51

**Published:** 2010-10-07

**Authors:** Stylianos Bournazos, Jacob Grinfeld, Karen M Alexander, John T Murchison, William A Wallace, Pauline McFarlane, Nikhil Hirani, A John Simpson, Ian Dransfield, Simon P Hart

**Affiliations:** 1 University of Edinburgh/Medical Research Council Centre for Inflammation Research, Queen's Medical Research Institute, Edinburgh, UK; 2 Centre for Cardiovascular Science, University of Edinburgh, Queen's Medical Research Institute, Edinburgh, UK; 3 Department of Radiology, Royal Infirmary of Edinburgh, Edinburgh, UK; 4 Department of Pathology, Royal Infirmary of Edinburgh, Edinburgh, UK; 5 Respiratory Medicine Unit, Royal Infirmary of Edinburgh, Edinburgh, UK; 6 Division of Cardiovascular and Respiratory Studies, Hull York Medical School/University of Hull, Castle Hill Hospital, Cottingham, UK

## Abstract

**Background:**

A significant genetic component has been described for idiopathic pulmonary fibrosis (IPF). The R131H (rs1801274) polymorphism of the IgG receptor FcγRIIa determines receptor affinity for IgG subclasses and is associated with several chronic inflammatory diseases. We investigated whether this polymorphism is associated with IPF susceptibility or progression.

**Methods:**

In a case-control study, we compared the distribution of FcγRIIa R131H genotypes in 142 patients with IPF and in 218 controls using allele-specific PCR amplification.

**Results:**

No differences in the frequency of FcγRIIa genotypes were evident between IPF patients and control subjects. However, significantly impaired pulmonary function at diagnosis was observed in HH compared to RR homozygotes, with evidence of more severe restriction (reduced forced vital capacity (FVC)) and lower diffusing capacity for carbon monoxide (DL_CO_). Similarly, increased frequency of the H131 allele was observed in patients with severe disease (DL_CO _< 40% predicted) (0.53 vs. 0.38; p = 0.03). Furthermore, the H131 allele was associated with progressive pulmonary fibrosis as determined by > 10% drop in FVC and/or > 15% fall in DL_CO _at 12 months after baseline (0.48 vs. 0.33; p = 0.023).

**Conclusions:**

These findings support an association between the FcγRIIa R131H polymorphism and IPF severity and progression, supporting the involvement of immunological mechanisms in IPF pathogenesis.

## Background

Idiopathic pulmonary fibrosis (IPF) is a non-neoplastic lung disease of unknown etiology and represents the most common clinical entity within the group of idiopathic interstitial pneumonias. The histological correlate of IPF is usual interstitial pneumonia (UIP), in which areas of interstitial fibrosis of various ages are interspersed with normal lung [1,2]. This histological pattern could be explained by repeated episodes of lung injury separated in time and place followed by an uncontrolled wound healing response that consequently leads to excessive fibrosis. Although for some fibrosing interstitial lung diseases the initiating lung injury is well defined and might include inhaled allergens, dust, or fibers [2], in IPF the cause of the initiating lung injury has not been identified. However, several lines of evidence support a role for immune complexes (antibody-antigen complexes) as mediators of lung injury in IPF that ultimately elicit a pro-fibrotic response [3]. Normally, immune complexes that are formed during an immune response are rapidly cleared by tissue-resident phagocytes. However, in many chronic inflammatory diseases elevated levels of circulating immune complexes as well as their deposition in tissues have been detected, which consequently promote tissue injury via activation of complement and engagement of leukocyte Fc receptors [4-6]. In the context of IPF, pulmonary fibrosis has been induced in animals following administration of immune complexes[3,7]. Secondly, lung fibrosis resembling IPF occurs in patients with autoimmune rheumatic diseases, including rheumatoid arthritis and scleroderma. Thirdly, immune complexes have been detected in the serum and the lung of patients with IPF [8-16].

IgG-containing immune complexes are recognized via Fcγ receptors, which are expressed predominantly by leukocytes. Substantial genetic variation in the form of single nucleotide polymorphisms (SNPs) has been described for Fcγ receptor genes, and their association with several chronic inflammatory and autoimmune diseases has been reported (reviewed in [17]). The R131H (rs1801274) substitution of FcγRIIa, a low-affinity IgG receptor that is widely expressed by diverse leukocyte types, maps to the receptor interface interacting with the Fc region of IgG and determines the affinity of FcγRIIa for human IgG subclasses [17]. The H131 but not the R131 variant is capable of interacting with IgG2 and has been previously shown to bind and mediate phagocytosis of IgG2-coated particles [18]. In the present study we investigated whether the FcγRIIa R131H polymorphism is associated with IPF disease susceptibility or progression.

## Methods

### Study Design and Subjects

In this case-control study, 142 IPF patients and 218 controls were recruited and the FcγRIIa R131H genotype was determined. For IPF patients, pulmonary function was assessed at baseline and repeated at intervals over 12 months to assess disease progression. IPF patients were categorized according to disease severity (DL_CO _< 40% predicted at presentation) and progression (reduction of ≥10% in FVC or ≥15% in DL_CO _in the first 12 months).

IPF (n = 142) was diagnosed in patients attending a specialist interstitial lung disease clinic according to the American Thoracic Society (ATS)/European Respiratory Society (ERS) international multidisciplinary consensus classification [2,19]. The baseline characteristics of the IPF cohort are summarized in Table 1. Bronchoalveolar lavage (BAL) and/or surgical lung biopsy were performed in cases for which a confident diagnosis on clinical, functional and radiological grounds was not possible. A consensus diagnosis was made in each case following joint review by two respiratory clinicians and a radiologist (and a pathologist for cases in which biopsy was performed). The control group (n = 218) comprised age-matched patients (n = 70) with a number of different acute or chronic lung pathologies other than an interstitial lung disease, ranging from asthma and seasonal influenza to pneumonia and lung cancer that were admitted to Edinburgh Royal Infirmary (mean age: 71.4 ± 10.2; female/male: 34/36) and healthy blood donors (n = 148; female/male: 65/83). No differences in the R131H genotype frequencies were evident between the control patient and healthy donor subgroups so these two subgroups were combined. All subjects were Caucasians. Ethical approval was obtained from the Lothian Research Ethics Committee (LREC/2002/4/65) and informed consent was obtained from all subjects.

**Table 1:** Baseline Pulmonary Function of the IPF Cohort

Patients, *n*	142
Age, *y (range)*	70 ± 8.8 (50-87)
Gender, *F/M (%)*	47/95 (33.1/66.9)

**FEV_1_**, *L*	2.16 ± 0.6
*% Predicted*	87.52 ± 20.0
**FVC**, *L*	2.76 ± 0.8
*% Predicted*	87.65 ± 19.6
**FEV_1_/VC**, *% predicted*	79.07 ± 9.9
**TLC**, *L*	4.30 ± 1.0
*% Predicted*	74.20 ± 15.3
**DL_CO_**, *ml/min/mmHg*	4.11 ± 1.4
*% Predicted*	52.75 ± 15.9
**K_CO_**, *ml/min/mmHg/L*	1.10 ± 0.3
*% Predicted*	82.47 ± 22.8

Values as mean ± SD. Abbreviations: IPF: idiopathic pulmonary fibrosis; DL_CO_: Diffusing capacity of the lung for carbon monoxide; K_CO_: DL_CO_corrected for lung volume.

Pulmonary function measurements were recorded at baseline (first radiologic evidence for IPF) and at 6 and 12 months (± 1 month) following diagnosis to assess disease progression in 121 patients with IPF. The remaining 21 patients were lost to follow up or were unfit to perform serial testing.

### FcγRIIa R131H Genotyping

Genomic DNA was extracted from 2 ml of peripheral venous blood using a QIAamp DNA Blood Midi Kit (Qiagen) following the manufacturer's instructions. For the determination of FcγRIIa R131H genotypes, PCR amplification reactions were performed using allele-specific PCR primers (Eurofins, MWG). For the R131 allele: forward: 5'-AAATCCCAGAAATTCTCAC**G**, reverse: 5'-CACTCCTCTTTGCTCCAGTG; For the H131 allele: forward: 5'-AAATCCCAGAAATTCTCAC**A**, reverse: 5'-CACTCCTCTTTGCTCCAGTG. Primers were designed based on the human DNA reference sequence (NCBI build 36.1). Nucleotides in bold are allele-specific and those underlined indicate nucleotides deliberately mismatched to the original gene sequences to increase primer specificity. PCR amplification reactions were performed in 50 μl volume, containing 200 μM dNTP, 1.5 mM MgCl_2_, 200 nM of each primer pair (forward and reverse), 2.5 U *Taq *DNA polymerase (Go*Taq *Flexi DNA Polymerase, Promega) in green 1× Go*Taq *Flexi buffer (Promega), and 1 μl of extracted genomic DNA (200-500 ng). PCR amplification conditions were 5 min at 94°C, followed by 30 cycles of 45 sec at 94°C, 45 sec at 54°C and 45 sec at 72°C and a final extension step of 5 min at 72°C. PCR products (269 bp) were analysed by 2% (w/v) agarose gel electrophoresis. The efficiency and specificity of the allele-specific PCR amplification was validated by direct sequencing using Applied Biosystems Big-Dye 3.1 chemistry on an Applied Biosystems model 3730 automated capillary DNA sequencer (College of Life Sciences, University of Dundee, UK). The number of samples analysed by direct sequencing corresponded to about 10% of the total analysed samples (n = 360) with a minimum of 10 for each tested genotype (RR, RH, HH). In all cases, the genotypes obtained from the sequencing-based method matched those obtained by PCR.

### Genetic and Statistical Analysis

Hardy-Weinberg equilibrium was assessed by a χ^2 ^test with one degree of freedom. Differences in the genotype and allele frequencies between control and IPF patients were analysed by the χ^2 ^or Fisher's exact tests. The statistical power (α = 0.05) of this study to detect differences in the minor (H131) allele frequency corresponding to odds ratio (OR) of 2 between control and IPF groups reached 98.8%. One-way analysis of variance (ANOVA) was used to test for differences in the mean values of quantitative variables, and where statistically significant effects were found post-*hoc *analysis using the Bonferroni test was performed. Unless otherwise stated, quantitative data are presented as mean ± SD and p < 0.05 was considered to be statistically significant. Data were analysed with GraphPad Prism software (Graphpad) and statistical power was calculated using the GraphPad StatMate software (Graphpad).

## Results

### FcγRIIa R131H polymorphism does not confer susceptibility to IPF

Both in the control and in the IPF cohort there was agreement between genotypes observed and those predicted by the Hardy-Weinberg equilibrium (controls: χ^2 ^= 0.008, p = 0.93; IPF: χ^2 ^= 1.83, p = 0.18). No significant differences in the distribution and frequency of the FcγRIIA R131H genotypes (RR, RH, HH) were observed between control subjects and IPF patients (RR: 0.36 for control vs. 0.37 for IPF; RH: 0.48 vs. 0.43; HH: 0.16 vs. 0.20; χ^2 ^= 1.38, *df*2, p = 0.50). Allelic frequency of R131 and H131 variants in IPF patients was comparable to those noted for the control group (R: control vs. IPF, 0.60 vs. 0.59; H: 0.40 vs. 0.41; p = 0.70, OR 1.07, 95% CI 0.79-1.44).

These data suggest there is no difference in the frequency of the FcγRIIa R131 polymorphism between control and IPF groups.

### The FcγRIIa H131 variant is associated with more severe disease at presentation

**Figure 1:**
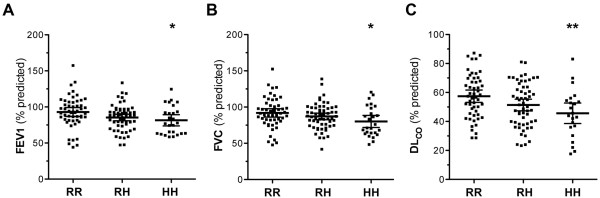
**H131 variant is associated with impaired pulmonary function at baseline**. (**A**) FEV1 (forced expiratory volume in 1 second), (**B**) FVC (forced vital capacity), and (**C**) DL_CO _(diffusing capacity of the lung for carbon monoxide) were determined in IPF patients and their association with FcγRIIa R131H genotypes was assessed. Data are presented as the mean percent predicted value ± 95% confidence intervals (CI). *p < 0.05 RR vs. HH, **p < 0.01 RR vs. HH.

We next compared the R131H genotype frequencies with pulmonary function measurements obtained at presentation. Homozygous carriers of the H allele had significantly lower FEV1 and FVC compared with RR homozygotes (Table 2; Figure 1A-B). In addition, substantially impaired pulmonary gas transfer was observed in H131 homozygous patients, as evidenced by significantly reduced levels of DL_CO _in these patients compared to RR homozygotes (Figure 1C). DL_CO _is a reliable guide to outcome and values of less than 40% of predicted are generally indicative of advanced disease[2]. Significantly increased frequencies of the HH and RH genotypes were evident in the IPF subgroup with DL_CO _< 40% vs. those with DL_CO _≥40% (< 40% DL_CO _HH: 0.25, RH: 0.56, RR: 0.19; ≥40% DL_CO _HH: 0.18, RH: 0.39, RR: 0.43; χ^2 ^6.1, *df*2, p = 0.04). In addition, the overall frequency of the H allele was increased in patients with DL_CO _< 40% compared to those with DL_CO _≥40% predicted (0.53 vs. 0.38; p = 0.03, OR 1.87, 95% CI 1.07-3.28). Homozygotes and heterozygotes of the H allele were associated with DL_CO _< 40% predicted (p = 0.01, OR 3.23, 95% CI 1.23-8.49). Collectively, these results indicate that the H allele is associated with reduced pulmonary function at presentation in IPF, which is indicative of more advanced disease.

**Table 2:** Baseline Pulmonary Function of IPF Patients According to FcγRIIa Genotypes

	FcγRIIa Genotype			
	RR	RH	HH	*P *value
Patients, *n*	53	61	28	
Age, *y*	70.1 ± 8.9	70.1 ± 8.9	69.8 ± 10.5	NS

**FEV_1_**, *L*	2.31 ± 0.6	2.12 ± 0.6	1.96 ± 0.5	0.037
*% predicted*	93.0 ± 22.0	85.7 ± 17.4	81.5 ± 19.4	0.031
**FVC**, *L*	2.92 ± 0.8	2.75 ± 0.8	2.43 ± 0.6	0.026
*% predicted*	91.9 ± 20.6	87.1 ± 17.4	80.1 ± 20.7	0.041
**FEV_1_/VC**, *% predicted*	79.7 ± 9.4	77.5 ± 10.2	81.3 ± 9.7	NS
**TLC**, *L*	4.47 ± 0.9	4.82 ± 1.1	3.94 ± 1.0	NS
*% predicted*	76.7 ± 14.0	72.9 ± 15.8	71.8 ± 16.5	NS
**DL_CO_**, *ml/min/mmHg*	4.49 ± 1.4	3.98 ± 1.2	3.54 ± 1.4	0.012
*% predicted*	57.4 ± 15.4	51.4 ± 15.0	45.7 ± 16.4	0.007
**K_CO_**, *ml/min/mmHg/L*	1.17 ± 0.3	1.07 ± 0.3	1.06 ± 0.3	NS
*% predicted*	87.6 ± 24.6	80.1 ± 20.5	76.6 ± 22.9	NS

Values as mean ± SD. P values (P) for RR vs. HH comparison. Abbreviations: NS: non-significant; IPF: idiopathic pulmonary fibrosis; DL_CO_: Diffusing capacity of the lung for carbon monoxide; K_CO_: DL_CO_corrected for lung volume.

### R131H polymorphism is associated with IPF disease progression

To further investigate IPF disease progression, serial lung function measurements for the first 12 months following baseline were obtained and the percent change in FVC and DL_CO _were determined.

A drop from baseline of ≥10% in FVC or ≥15% in DL_CO _in the first 6-12 months is associated with higher mortality and with a more aggressive form of IPF[2,20-22]. Therefore, patients were categorized into either progressive (n = 49) or non-progressive (n = 72) groups and their association with the FcγRIIa R131H genotypes was investigated. Significant skewing in the distribution of the R131H genotypes (RR, RH, and HH) was noted between progressive and non-progressive groups (Table 3). In particular, in the progressive group there was higher frequency of the HH genotype (0.29 vs. 0.11) as well as decreased RR genotype frequency (0.33 vs. 0.44; χ^2 ^= 6.13, *df*2, p = 0.047) compared to the non-progressive group. In addition, the frequency of the H allele was increased in the progressive group compared to the non-progressive group (0.48 vs. 0.33; p = 0.023, OR 1.84, 95% CI 1.09-3.12).

**Table 3:** Genotype Frequencies and Pulmonary Function of Progressive and Non-progressive Subgroups of IPF Patients

		Progressive Group	Non-Progressive Group
Patients, *n*		49	72
Age, *y (range)*		70.51 ± 9.3	69.95 ± 8.9
Gender, *F/M (%)*		12/37 (24.5/75.5)	26/46 (36.1/63.9)
FcγRIIa Genotypes, *n (%)*	**RR**	16 (32.7)	32 (44.4)
	**RH**	19 (38.8)	32 (44.4)
	**HH**	14 (28.6)	8 (11.1)
		χ^2 ^= 6.13, *df*2, p = 0.047	
FcγRIIa Alleles, *n (%)*	**R**	51 (52.0)	96 (66.7)
	**H**	47 (48.0)	48 (33.3)
		p = 0.023, OR 1.84, 95% CI 1.09-3.12	
**FEV_1_**, *L (% predicted)*		2.29 ± 0.6 (90.3 ± 20.9)	2.19 ± 0.6 (89.2 ± 18.8)
**FVC**, *L (% predicted)*		2.81 ± 0.7 (86.8 ± 21.1)	2.86 ± 0.8 (91.7 ± 17.8)
**FEV_1_/VC**, *% predicted*		81.96 ± 9.9	77.05 ± 9.5
**TLC**, *L (% predicted)*		4.33 ± 1.0 (71.5 ± 15.6)	4.43 ± 1.0 (76.8 ± 13.5)
**DL_CO_**, *ml/min/mmHg (% predicted)*		4.13 ± 1.3 (52.2 ± 16.3)	4.18 ± 1.3 (53.8 ± 14.3)
**K_CO_**, *ml/min/mmHg/L (% predicted)*		1.08 ± 0.3 (82.7 ± 22.8)	1.10 ± 0.3 (81.7 ± 22.8)

Disease progression groups were determined based on changes in FVC or DL_CO_. Progressive group displayed a ≥10% decrease in FVC and/or a ≥15% decrease in DL_CO _12 months after baseline measurements. Values as mean ± SD. Abbreviations: IPF: idiopathic pulmonary fibrosis; DL_CO_: Diffusing capacity of the lung for carbon monoxide; K_CO_: DL_CO_corrected for lung volume.

**Figure 2:**
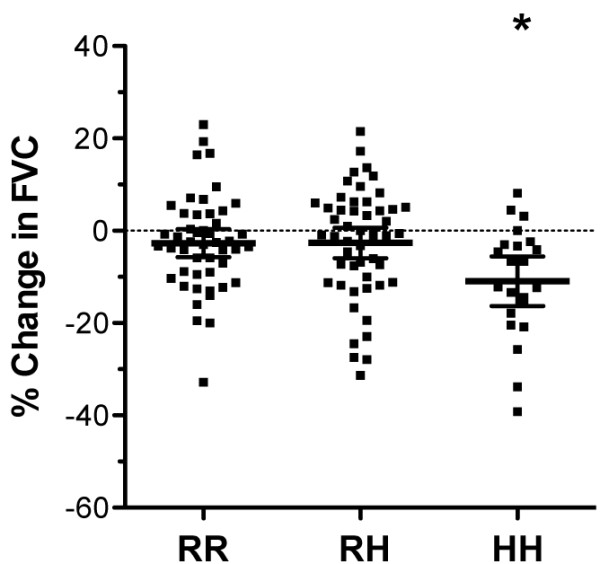
**R131H polymorphism is associated with disease progression**. Serial lung function measurements were obtained for 121 IPF patients 12 months following baseline to assess disease progression. FVC (forced vital capacity) displayed a significant fall in HH, but not RR or RH patients. Data are presented as the mean percent change in actual values 12 months following baseline ± 95% confidence intervals (CI). *p < 0.01 RR vs. HH.

Furthermore, a significant reduction in FVC was observed in the first 12 months in HH homozygotes, compared to homozygous and heterozygous carriers of the R allele (Figure 2). In contrast, no major change in DL_CO _was evident between the three R131H genotypes, despite the lower baseline DL_CO _values observed in HH patients (data not shown). In summary, all these findings suggest that the FcγRIIa R131H polymorphism represents a genetic risk factor for IPF disease progression.

## Discussion

The current model of IPF pathogenesis suggests a dysregulated wound-healing response with minimal inflammation in response to alveolar epithelial damage. Although the cause of epithelial injury is unknown, immunological determinants in IPF pathogenesis should be considered [23]. In the present study, we have demonstrated that the H131 variant of FcγRIIa is associated with disease severity at presentation and progression over the subsequent 12 months. In particular, increased frequency of the H131 allele was observed in patients with more advanced disease (DL_CO _< 40% predicted). Similarly, HH homozygotes displayed significantly impaired baseline pulmonary function compared to RR homozygotes, with evidence of more severe restriction and reduction in gas transfer. In addition, significant association of this allele was evident in IPF patients with progressive disease (> 10% drop in FVC and/or > 15% in DL_CO_).

Our data also suggest that lung function is dependent on the number of H alleles. In particular, as illustrated in Figure 1A-C and Table 2, baseline pulmonary function seemed to correlate with H alleles frequency in each patient. For example, more severe restriction and impaired gas transfer was evident in HH homozygotes compared to RH heterozygotes, and accordingly, in RH heterozygotes compared to RR homozygotes. This observation suggests a dose-dependent effect for the H allele on pulmonary function and therefore in disease manifestation. Similarly, a dose response relationship has been previously demonstrated for the R131 allele and SLE [24]. Collectively, our findings suggest that the R131H polymorphism is an additional determinant that influences IPF severity and progression.

Previous studies on the FcγRIIa R131H polymorphism have provided information about the functional consequences of this polymorphism. Association of the H131 variant has also been previously reported for a number of chronic inflammatory disorders, including periodontitis and Guillain-Barré syndrome [25-27], possibly due to the increased capacity of this variant for efficiently recognising IgG2. Since FcγRIIa is expressed by diverse leukocyte types, including macrophages and neutrophils which are typically observed in the BAL fluid of IPF patients [2,17], the R131H polymorphism is likely to influence IgG-mediated effector responses in these cell types. Indeed, engagement of FcγRIIa with IgG-containing immune complexes initiates a number of leukocyte effector responses including antibody-dependent cellular cytotoxicity, phagocytosis, production and release of proteolytic enzymes including matrix metalloproteinases, and generation of reactive oxygen and nitrogen intermediates[28]. Ensuing damage to the alveolar walls and pulmonary interstitium could lead to fibroblast activation and aberrant deposition of fibrotic tissue which is characteristic of IPF. Accelerated pulmonary damage and fibrosis would result in more severe disease pathology and progression, as evidenced by more severe lung restriction (reduced FEV_1 _and FVC) and reduced gas transfer (DL_CO_), which in the present study are associated with the H131 allele.

Although the ancestral allele for the R131H polymorphism is the H allele, in the present study the R allele was more prevalent than the H allele. Since the H131 allele represents the sole human leukocyte Fc receptor allotype capable of binding to IgG2 it might have been subject to strong evolutionary forces which shaped R131H genotype distribution in order to confer protection from a number of autoimmune and chronic inflammatory disorders. This assumption is strengthened by the fact that the frequency of the R131H genotypes varies greatly between different ethnic groups, especially Caucasians and Asians, suggesting the presence of different evolutionary forces in different populations [29,30]. It should be noted that the genotype frequencies observed in this study are in accordance with those reported in several previously published studies in Caucasian populations [29,31].

Previous studies provide evidence for a role for immune complexes in IPF disease pathogenesis, either through initiation of local inflammatory responses or direct mediation of lung injury [3,7-12,14-16,32]. Further evidence for a role of immune complexes in IPF pathogenesis is provided by the association of IPF susceptibility with polymorphisms in the complement receptor 1 (CD35) gene that is involved in the clearance of circulating immune complexes [33]; however, additional studies failed to confirm such association in other ethnic groups[34,35]. IgG-mediated production and release of proinflammatory cytokines by FcγRIIa-expressing leukocytes could amplify local inflammatory responses, leading to increased leukocyte infiltration [28]. A number of pro-inflammatory cytokines have been detected in the BAL fluid of IPF patients, including IL-1β, IL-4, IL-8, IL-13, TNF-α, TGF-β. Elicitation of the production of key fibrogenic molecules would accelerate fibrosis and disease progression[36-39]. Several reports describe associations of IPF with genes involved in proinflammatory pathways, including cytokines, chemokines and their corresponding receptors, and genes involved in tissue repair and fibrogenesis [40-49].

## Conclusions

In summary, we have here reported that the FcγRIIa R131H polymorphism is associated with IPF severity and progression, providing additional support for the role of immunological mechanisms in the development of IPF. This not only supports existing evidence on the pathogenic potential of immune complexes, but also reveals a novel role of Fcγ receptors in IPF disease progression. Given the relatively small size of the IPF cohort investigated here, additional studies on other IPF patients and different ethnic populations should be undertaken to further strengthen the observed association. Collectively, our results along with previous genetic association studies on IPF suggest that multiple genetic factors in combination with other environmental and immunological triggers can influence predisposition as well as disease progression. Identification of genetic variants that affect susceptibility or progression of IPF by altering the activity of molecules involved in inflammatory and pro-fibrotic pathways provides novel insights into the precise pathogenic mechanisms underlying IPF.

## Competing interests

The authors declare that they have no competing interests.

## Authors' contributions

SB participated in study design, performed DNA extraction and genotyping, analyzed genotypes and pulmonary function data, and drafted the manuscript; JG recruited patients and participated in DNA extraction and genotype determination; KMA developed and optimized the allele-specific PCR assay; JTM analyzed radiological data and established diagnosis; WAW performed histopathologic analysis and established diagnosis; PMcF recruited study subjects, collected blood samples, and kept records of diagnostic data; NH and AJS recruited IPF patients, established diagnosis, and obtained pulmonary function data; ID provided intellectual input, designed the study and drafted the manuscript; SPH designed and supervised the study, recruited subjects and established diagnosis, provided intellectual input and drafted the manuscript. All authors read and approved the final manuscript.

## Pre-publication history

The pre-publication history for this paper can be accessed here:

http://www.biomedcentral.com/1471-2466/10/51/prepub
